# Mitofusin 2: from functions to disease

**DOI:** 10.1038/s41419-017-0023-6

**Published:** 2018-02-28

**Authors:** Riccardo Filadi, Diana Pendin, Paola Pizzo

**Affiliations:** 1 Department of Biomedical Sciences, University of Padova, Padova, Italy; 2 Neuroscience Institute, National Research Council (CNR), Padova, Italy

## Abstract

Mitochondria are highly dynamic organelles whose functions are essential for cell viability. Within the cell, the mitochondrial network is continuously remodeled through the balance between fusion and fission events. Moreover, it dynamically contacts other organelles, particularly the endoplasmic reticulum, with which it enterprises an important functional relationship able to modulate several cellular pathways. Being mitochondria key bioenergetics organelles, they have to be transported to all the specific high-energy demanding sites within the cell and, when damaged, they have to be efficiently removed. Among other proteins, Mitofusin 2 represents a key player in all these mitochondrial activities (fusion, trafficking, turnover, contacts with other organelles), the balance of which results in the appropriate mitochondrial shape, function, and distribution within the cell. Here we review the structural and functional properties of Mitofusin 2, highlighting its crucial role in several cell pathways, as well as in the pathogenesis of neurodegenerative diseases, metabolic disorders, cardiomyopathies, and cancer.

## Facts

- MFN2, an outer mitochondrial membrane GTPase, is critical for mitochondrial fusion, which in turn affects mitochondrial dynamics, distribution, quality control, and function.
- MFN2 modulates ER−mitochondria tethering.
- Several mutations in the *Mfn2* gene, particularly in the GTPase domain, are associated to CMT2A.
- Altered MFN2 expression is associated with different pathological conditions.

## Open questions

- Which are the common/specific functions of MFN1 and MFN2?
- What is the molecular mechanism by which MFN2 modulates ER−mitochondria tethering?
- Which MFN2 function/s impairment is mainly implicated in CMT2A onset?
- How does MFN2 depletion cause ER stress?
- Is the altered MFN2 expression, described in different disorders, causally linked to their pathogenesis?

## Introduction

In most cells, mitochondria are organized in a tubular, dynamic network that undergoes continuous remodeling. Indeed, these organelles are mobile and can either divide (via fission processes), forming separated entities, or collide and fuse (via fusion), forming a more continuous network. In specific circumscribed regions, mitochondria are in close contact with other organelles, notably the endoplasmic reticulum (ER), although without fusing with them. Interestingly, ER−mitochondria contact sites favor mitochondrial constrictions and consequent fission^1^. Increasing evidence suggests that mitochondrial morphology is strictly connected to organelle functionality and, importantly, it can quickly change in response to cell conditions. A fused, continuous network is associated to a higher adenosine triphosphate (ATP) production, likely due to optimized exchanges of metabolites and mitochondrial DNA (mtDNA) within their matrix. This is observed, for instance, upon starvation^2,3^, when substantial ATP supply becomes critical for cell survival. On the contrary, mitochondrial fragmentation has been associated to a reduced respiration, frequently observed in cancerous cells in which the so called “Warburg effect” takes place (recently reviewed in ref. 4). Another aspect influenced by fission/fusion balance, particularly important for neuronal cells, is mitochondria transport and distribution along axons. The isolation of single mitochondria from the main network by fission is essential for their transport by the motor protein apparatus.

Given the importance of mitochondrial morphology in the regulation of multiple cell functions, and the potential connection with several pathologies, the importance of a detailed knowledge of the molecules/mechanisms that govern mitochondrial fusion and fission processes appears clear. While the list of the proteins controlling these opposite events is nowadays established, more debated are the exact molecular mechanisms through which they exert their activity.

Briefly, mitochondrial fission is mediated by the recruitment of the cytosolic GTPase dynamin 1-like protein (DNM1L/Drp1) on the outer mitochondrial membrane (OMM)^5^, through interaction with mitochondrial fission factor (Mff)^6^, Mid51, Mid49^7^, and perhaps fission 1 (Fis1)^8^ (reviewed in ref. 9). Mitochondrial fusion is unique, compared to other intracellular fusion events, because it involves two membranes, i.e., the OMM and the inner mitochondrial membrane (IMM), that must be rearranged in a coordinated manner in order to maintain organelle’s integrity. In particular, the OMM GTPases Mitofusin 1 (MFN1) and Mitofusin 2 (MFN2) are responsible for the fusion process of the OMM^10^, while optic atrophy 1 (OPA1) mediates IMM fusion^11^. A detailed discussion on these topics, as well as their relationship with pathology, is beyond the scope of this contribution and the interested readers are referred to some recent reviews^12–14^. Here, we limit ourselves to discuss the more recent findings on one of the proteins mediating OMM fusion, MFN2. We will briefly summarize the structural properties, the proposed mechanisms of action, and the functional roles of MFN2. In particular, we will review the link between MFN2 alterations and the onset/progression of different diseases/pathological conditions.

## MFN2: structural insights

In mammals, MFN1 and MFN2 are homologs proteins that belong to the large family of mitochondrial transmembrane GTPases, characterized firstly in *Drosophila melanogaster* as “fuzzy onions” (Fzo) protein^15^. In eukaryotes, this family has homologs from yeast to humans^16^, with structural properties conserved among different species.

**Fig. 1:**
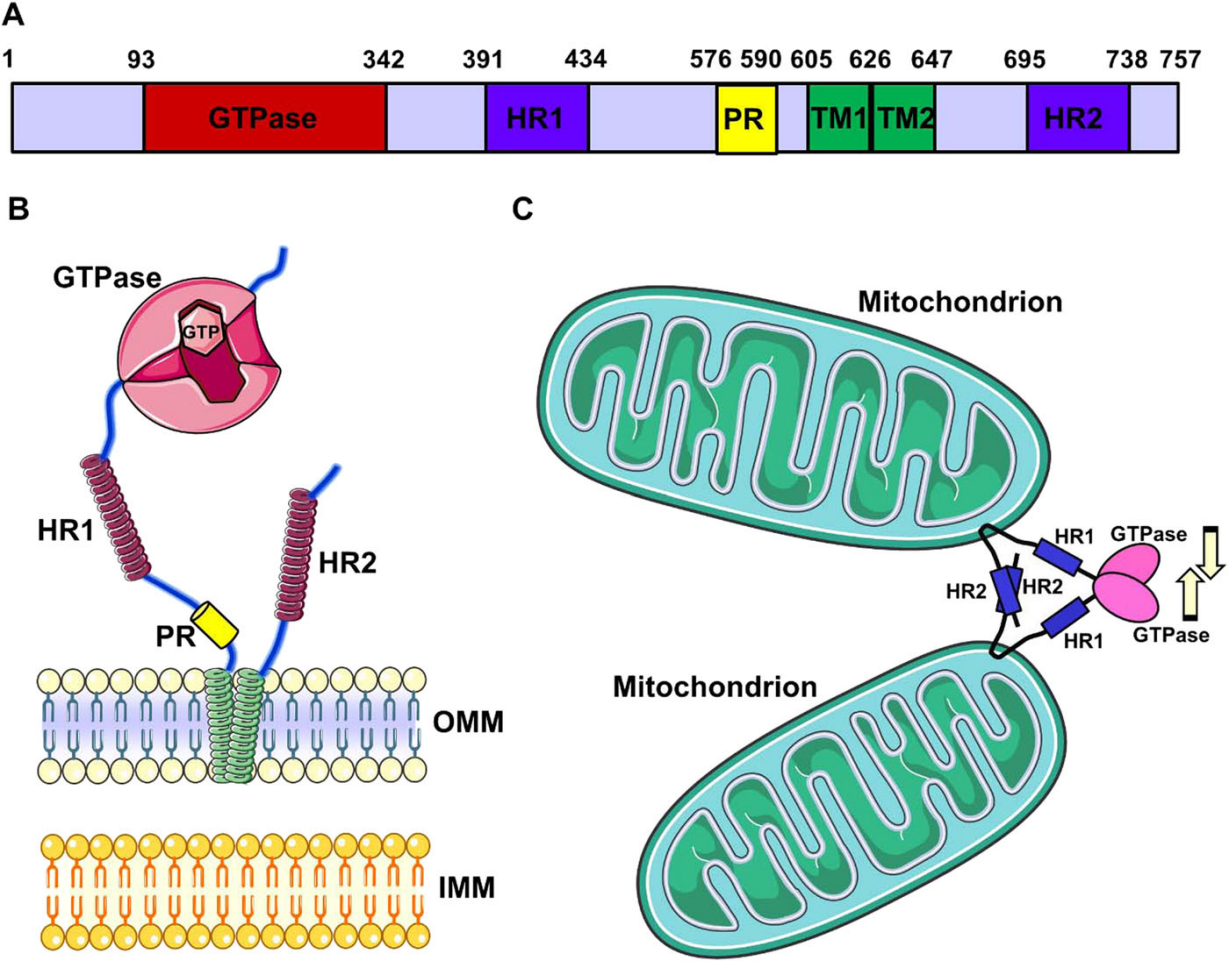
**MFN2 structure** **a** The scheme represents the linear structure of MFN2. Note the large N-terminal GTPase domain, followed by the HR1-domain, the PR domain, the two TM domains, and the C-terminal HR2 domain. The numbers above indicate the initial and the terminal amino acids of the corresponding domains. **b** The cartoon represents MFN2 topology, with two very close TM domains crossing the OMM (green helices) and the indicated cytosolic portions. Note the GTPase domain with two GTP-binding pockets. **c** Scheme of the OMM-fusion activity of MFNs. Tethering is mediated by the interaction between HR2 domains belonging to MFNs on opposite OMM. Recent data suggest that dimerization of the GTPase domains, as well as a power stroke due to GTP hydrolysis, may be important for fusion (see text for details)

In particular, mammalian MFN1 and MFN2 are highly similar proteins (~80% similarity in humans), consisting of 737 and 757 amino acids, respectively. They are endowed with a large, cytosolic, N-terminal GTPase domain, sequentially followed by a spacer, a first coiled-coil heptad-repeat (HR1) domain, a spacer, two very close transmembrane domains (TM) crossing the OMM, a spacer and a second, C-terminal heptad-repeat domain (HR2) (Fig. 1). Notably, between HR1 and the TM domains, only MFN2 possesses a proline-rich (PR) domain, likely responsible for specific protein−protein interactions.

MFNs have been shown by electron microscopy (EM) to accumulate in contact regions between adjacent mitochondria^17–19^, supporting their role in mitochondrial fusion. Though the exact molecular mechanisms through which MFNs mediate this process is still not completely understood, seminal studies revealed that MFNs, spanning from the OMM of two opposing mitochondria, physically interact in *trans*, by formation of antiparallel dimers between their HR2 domains^20^ (Fig. 1). This interaction, whose structure has been resolved, consists of a 95 Å coiled-coil region that tethers two mitochondria, but is insufficient to complete their fusion. Indeed, expression of MFN1 deprived of its N-terminal GTPase domain induces, in an HR2-dependent manner, mitochondria aggregation into typical structures, in which organelles are densely packed with a uniform gap of ~15 nm between opposing OMM^20^. This gap is likely covered by a combination of the HR2−HR2 dimer with the spacers located between HR2 and TM domains (Fig. 1). Thus, HR2 domains are important for the initial tethering between adjacent mitochondria, while the GTPase domain is likely critical for fusion completion. Interestingly, a different GTPase activity has been documented for MFN1 and MFN2^21^ and this is likely responsible for the different roles they play in mitochondrial fusion (see below).

Two recent additional studies, reporting MFN1 structure^22,23^, as well as the comparison with the properties of a cyanobacteria homologous of MFNs (the bacterial dynamin like protein, BDLP^24,25^), highlighted the mechanisms of OMM fusion (reviewed in ref. 26). Briefly, in addition to the HR2−HR2 interaction, the dimerization *in trans* of GTPase domains allows the initial tethering, and a GTP hydrolysis-dependent power stroke should be then responsible for pulling the membranes together, allowing their fusion (Fig. 1). This model has been proposed for MFN1, but is possibly (and likely) applicable to MFN2 as well. Interestingly, a very recent paper suggested the existence of two distinct and dynamic conformational states of mammalian MFNs^27^. According to this model, in the resting state MFNs are tethering-non-permissive, because of intramolecular, antiparallel HR1−HR2 interactions and a strict adherence of the globular, GTPase domain to the OMM. In the tethering-permissive state, on the contrary, the destabilization of the intramolecular HR1−HR2 interaction allows the HR2 domain to extend into the cytosol, where it can encounter and bind HR2 domains of MFNs in the opposing membrane, mediating tethering as previously suggested^20^. The flexing of each MFNs HR2 domain is responsible for retraction of tethered mitochondria, reducing the gap between them and allowing a GTPase-dependent fusion of the opposing membranes. Interestingly, a TAT-peptide-mediated strategy has been demonstrated to be effective in destabilizing the tethering-non-permissive HR1−HR2 interaction, correcting the fusion defects observed with some MFN2 mutants linked to Charcot−Marie−Tooth disease type 2A (CMT2A, see below)^27^.

## MFN2: functional roles

A pivotal in vivo study by Chan’s group revealed that both MFNs are essential for embryonic development^28^. Indeed, deletion of either *Mfn1* or *Mfn2* in mice is lethal during midgestation. While heterozygous animals are fully viable and fertile, the homozygous die, with a specific impairment in the formation of placenta in MFN2-knock-out (KO), but not in MFN1-KO mice. The double KO for both MFN1 and MFN2 is lethal at an even earlier stage of embryonic development. Interestingly, conditional inactivation of *Mfn1* or *Mfn2* alleles after placentation revealed that, while *Mfn1* ablation is fully compatible with life through adulthood, *Mfn2* ablation severely impairs *cerebellum* development, with early movement defects in newborn mice that succumb before P17^29^. In conditional *Mfn1*^−^*/*^*−*^ mice, however, the loss of just one *Mfn2* allele is lethal. These results suggest that MFN1 and MFN2 play partially redundant and distinct functions, depending on the developmental state. In this scenario, a further complication is provided by the fact that, though MFN1 and MFN2 are ubiquitously expressed, they display different levels of expression among tissues. While in liver, kidney, and adrenal glands they are expressed at comparable levels, in testis and heart MFN1 is predominant. Interestingly, in the brain, MFN2 is abundantly expressed, but MFN1 only marginally^19^. Thus, it is tempting to speculate that, in addition or alternatively to a slightly different molecular function, the tissue-specific phenotype induced by MFNs ablation could be due to a different expression pattern: being MFN2 largely predominant in the brain, it is not surprising that its ablation induces cerebellar-specific impairments.

### Mitochondrial fusion

**Fig. 2:**
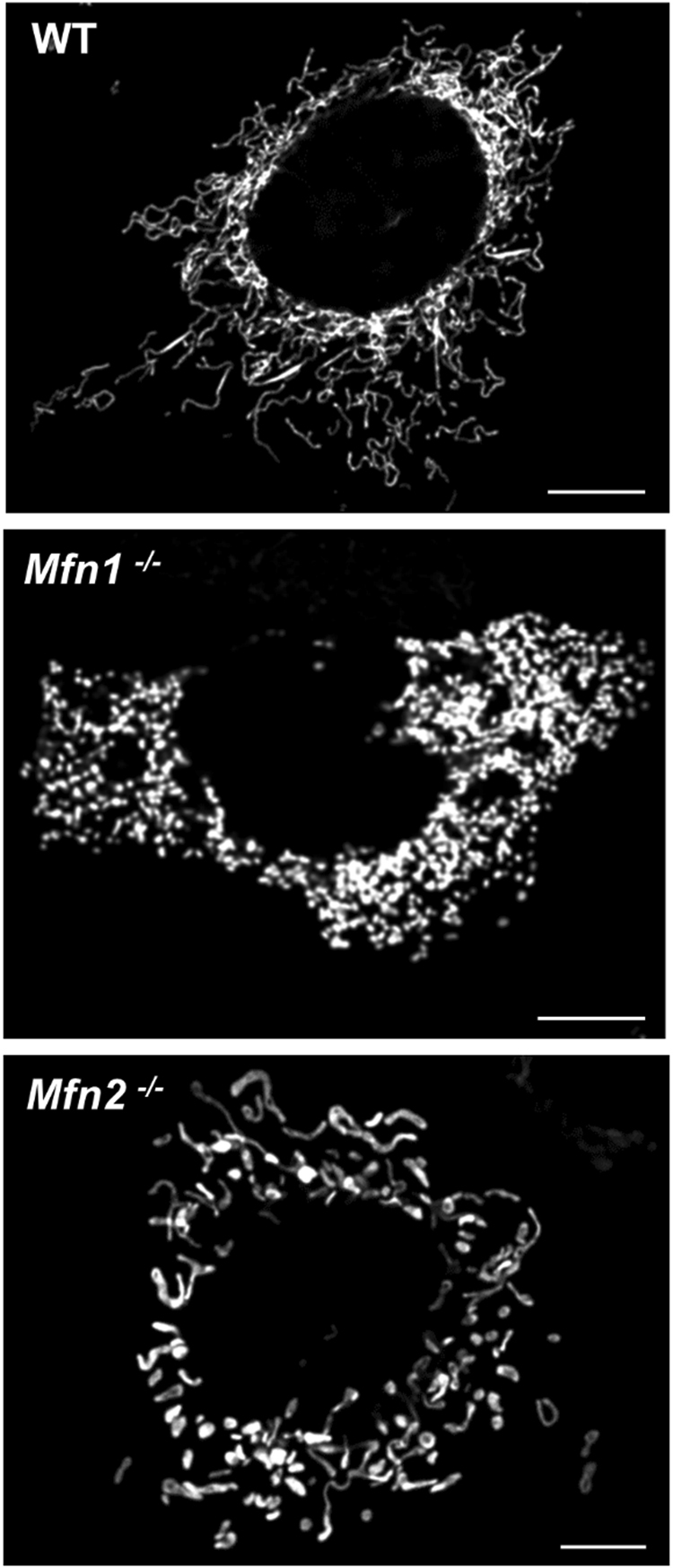
**Effects of MFNs ablation on mitochondrial morphology** Representative confocal microscopy images of WT, *Mfn1*^−/−^ and *Mfn2*^−/−^ MEF cells, expressing a mitochondrial matrix-targeted RFP. Scale bar: 5 μm. Note the fragmented mitochondrial network in MFNs ablated cells, with creation of small spheres in *Mfn1*^−/−^ MEFs and of more enlarged structures of variable size in *Mfn2*^−/−^ MEFs

Both MFN1- and MFN2-deficient cells display an aberrant mitochondrial morphology, with a clear fragmentation of the network^28^. However, their ablations lead to characteristic and promptly distinguishable morphologies (Fig. 2). While MFN1-KO induces a severe mitochondrial fragmentation, with formation of small spheres uniform in size, MFN2-KO cells display mitochondrial spheres or ovals of widely different size, some of them showing a diameter several fold larger than that of wild type (wt) mitochondrial tubules (ref. 28; but see also ref. 30). Interestingly, the overexpression of MFN1 in MFN2-KO, or of MFN2 in MFN1-KO cells, is able to partially rescue the lack of the partner protein and promote mitochondrial fusion^28^, further highlighting a certain grade of redundancy. The overexpression of either MFN1 or MFN2 in a wt context has been reported to induce mitochondrial aggregation and collapse in the perinuclear region^17,19,30^. The reasons for this paradoxical phenotype are unknown. The exaggerated MFNs-induced docking/tethering of adjacent mitochondria could be caused by the lack of a parallel increased activity of still unknown additional factors that may be essential for the completion of the fusion process. Alternatively, a fusion non-permissive state of MFNs (see above and ref. 21) could be responsible.

### ER−mitochondria contacts

**Fig. 3:**
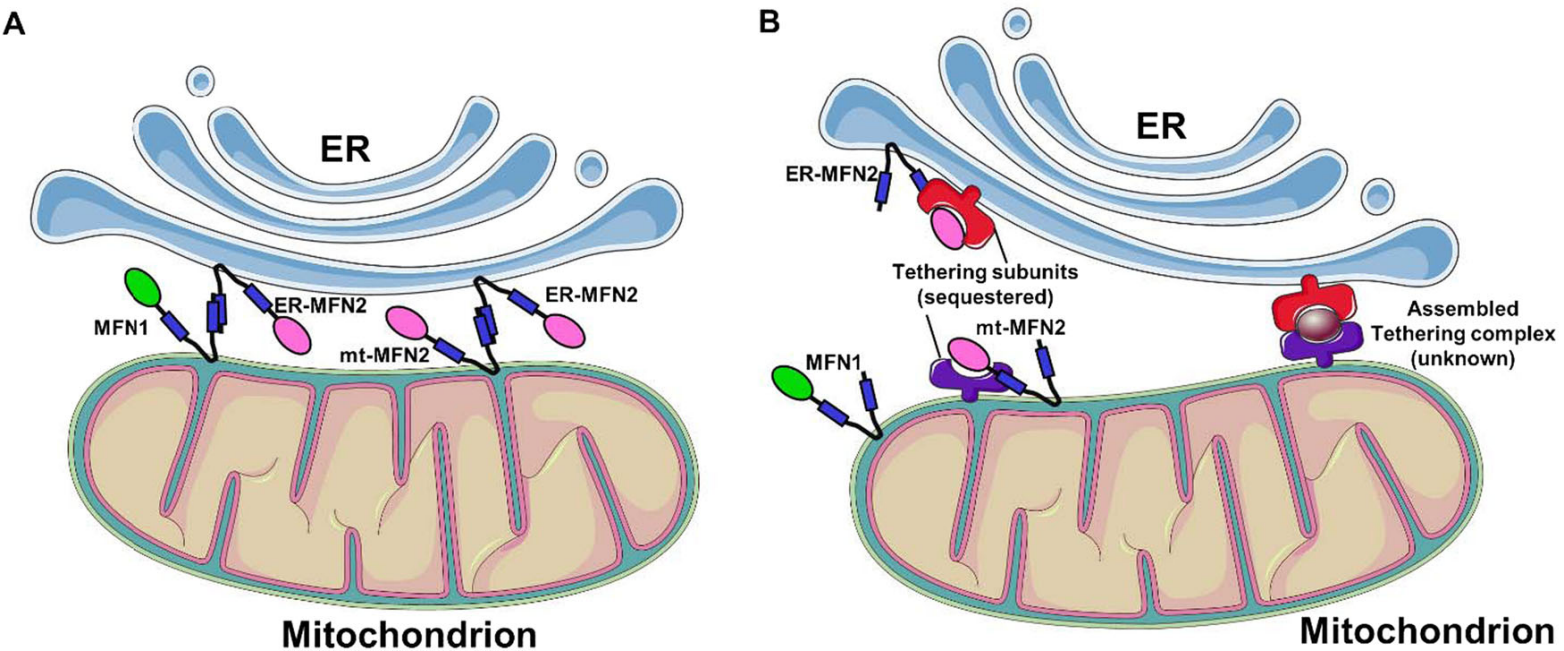
**Alternative models for MFN2-mediated ER−mitochondria tethering** **a** The scheme represents the classical view of MFN2 as a positive modulator of ER−mitochondria juxtaposition. In this model, MFN2 on ER membrane engages MFN2 or MFN1 on OMM, mediating the tethering between the two organelles. **b** The scheme represents the model of MFN2 as a negative modulator of ER−mitochondria juxtaposition. According to this view, MFN2 on both the ER and the OMM interacts with and sequesters still unknown tethering subunits (left), hindering their assembling into a functional tethering complex (represented on the right)

In addition to its undisputed role in mitochondrial fusion, MFN2 has been suggested to be a key regulator of ER−mitochondria juxtaposition, though its exact function in this inter-organelle interplay still remains matter of intense debate. Notably, and differently from MFN1, a small fraction of MFN2 has been observed to be located in ER membranes, particularly in the so-called ER mitochondria-associated membranes (MAM), i.e., ER regions juxtaposed to the OMM^31^. At this level, based on its function in the tethering/fusion of adjacent mitochondria, MFN2 has been classically proposed to mediate ER−mitochondria tethering, by engaging in homo- or heterotypic interactions MFN2 or MFN1 located in the OMM^32^. After this initial study, several processes known to be regulated, or to directly take place at MAM, such as autophagosomes formation, were reported to be modulated by the presence of MFN2. This indeed created consensus on the validity of the initial model, though in most cases the effects of MFN2 on ER−mitochondria juxtaposition were just assumed and not directly evaluated (see e.g. refs.^33–35^ and for more details ref. 36). However, more recent studies challenged the ER−mitochondria tethering activity of MFN2, based on the finding (obtained by quantitative EM) that the averaged percentage of OMM in contact with the ER is actually increased and not decreased in MFN2-KO cells, or upon acute MFN2 downregulation^30,37^. The apparent contradiction between the increased juxtaposition (retrieved by quantitative EM in MFN2 ablated cells^30,37^ and indirectly confirmed by additional studies^38–41^) and the reduced ER−mitochondria co-localization (originally observed by confocal microscopy^32^, but notably also confirmed in the challenging papers^30,37^), was solved by the demonstration that the latter result is an artifact, due to marked changes in mitochondrial morphology induced by MFN2-downregulation^30^. Indeed, whenever only the perimeter of mitochondria, and not their entire volume, was considered, an increased co-localization with the ER was retrieved also by confocal microscopy. Moreover, an overall increased ER−mitochondria coupling upon MFN2-downregulation was suggested also by a number of functional assays, particularly by a favored ER to mitochondria Ca^2+^ transfer^30^. Very recently, a paper from Scorrano’s group claimed to confirm their original findings^32^ on the basis of a number of techniques, including quantitative EM, Ca^2+^ transfer assays and Forster resonance energy transfer (FRET)-based measurements of organelles vicinity^42^, but the validity of some of these experiments has been questioned^43^. A detailed discussion on this controversy, reported in Fig. 3, is beyond the scope of this review and the interested readers are referred to the original articles and to a recent contribution^36^. It is our biased opinion, however, that while it is undisputed that MFN2 plays an important role in the modulation of ER−mitochondria tethering, the finding, in MFN2-KO cells, of either an increased^30,37^ or, at least, a still largely present^32,42^ ER−mitochondria connection, inevitably suggests that this protein is not bone fide essential in the formation/maintenance of this inter-organelles tethering. Further investigations will be necessary to more accurately evaluate the impact of MFN2 in this key intracellular pathway.

### ER stress

**Fig. 4:**
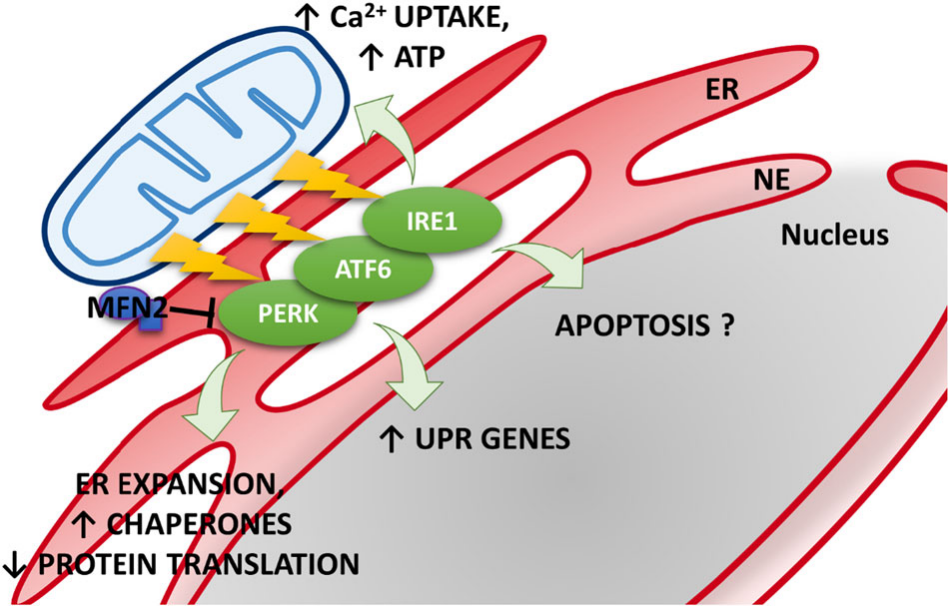
ER stress induced by MFN2 depletion *Mfn2* ablation induces the activation of UPR proteins located in ER membranes. In particular, MFN2 has been proposed as an upstream modulator of PERK that under basal conditions maintains the kinase inactive. Once activated, UPR works by expanding the ER, upregulating chaperones and inhibiting protein translation. Mitochondria display higher Ca^2+^ uptake and ATP production to support the higher energy demand. It is still unclear whether *Mfn2* ablation induces an increase or decrease of ER stress-induced apoptosis

Another key role played by MFN2, linked to its involvement in ER−mitochondria association, refers to the ER stress response. The ER stress response constitutes a process triggered by a variety of conditions that disturb protein folding within the organelle, e.g., protein synthesis impairment, Ca^2+^ imbalance, and redox capacity defects. During evolution, cells have developed a complex signal transduction mechanism, the unfolded protein response (UPR), that aims at clearing unfolded proteins and restoring ER homeostasis. The specialized proteins PERK (protein kinase RNA (PKR)-like ER kinase), IRE1 (inositol-requiring protein 1), and ATF6 (activating transcription factor 6) located in ER membranes can detect unfolded proteins accumulation and activate specific signaling pathways^44^. UPR works by expanding the ER, upregulating chaperones and causing a temporary stop of the translation (Fig. 4). This phase is accompanied by the strengthening of ER−mitochondria contact sites, reasonably in order to support the high energy demand of ER stress-induced transcriptional machinery^45^. On their side, mitochondria display an increase in transmembrane potential and oxygen consumption, higher Ca^2+^ uptake, and ATP production. When ER stress cannot be reversed, cellular functions deteriorate, often leading to mitochondria-mediated cell death^46^. ER−mitochondria contact sites plays a fundamental role in the ER stress response, both in the first phase, when high energy is demanded, and during apoptotic cell death^47^. Indeed, several studies have corroborated a direct link between changes in MAM components, deregulated Ca^2+^ transfer and lipid composition and apoptotic sensitivity during ER stress^44^. In particular, MFN2 ablation have been shown to induce ER stress in different models, from mouse embryonic fibroblasts (MEFs)^48,49^ and cardiac myocytes^49,50^ to mouse liver^51^ and drosophila tissues^52^. In particular, the work of Zorzano and colleagues reported the induction of UPR mediators in MFN2-deficient MEFs under basal or ER stress conditions^51^. Unexpectedly, PERK silencing rescued some of the phenotypes caused by *Mfn2* ablation, suggesting that MFN2 is an upstream modulator of PERK that under basal conditions maintains the kinase inactive (Fig. 4). Accordingly, the authors showed that MFN2 physically interacts with PERK. The work suggested that PERK controls mitochondrial morphology and function, as well as oxidative stress in cells and proposed that *Mfn2* ablation-dependent tissue alterations are, at least in part, the result of enhanced PERK activity. These cells showed reduced activation of apoptosis and autophagy^48^. These results, although in line with other findings^53^, are in disagreement with Walsh and co-authors, reporting that *Mfn2* ablation in MEFs or in mice exacerbated ER stress-induced apoptosis^49^. ER stress appears also a component of the complex phenotype caused by ablation of the single drosophila ortholog of *Mfns* (Mitochondrial assembly regulatory factor, *Marf*): ubiquitous, neuron- or muscle-specific *Marf* ablation was lethal for flies, altering mitochondrial and ER morphology and triggering ER stress. Pharmacological reduction of ER dysfunction ameliorates the functional and developmental defects of flies lacking Marf, correcting also ER shape^52^. Finally, accumulating evidence implicates prolonged ER stress in the development and progression of various diseases, and a specific role for MFN2 has been proposed in some of these pathogenic processes (see below).

### Mitophagy

The proteins regulating mitochondrial dynamics are usually closely involved in the mitochondria quality control process, known as mitophagy. Two important mediators of this process are PINK1 and the E3 ubiquitin-protein ligase parkin. PINK1 selectively accumulates on the OMM of depolarized mitochondria, while cytosolic parkin ubiquitinates proteins targeted for degradation. In particular, upon mitophagy induction, such as during carbonyl cyanide 4-(trifluoromethoxy)phenylhydrazone (FCCP)-mediated mitochondrial depolarization, parkin ubiquitinates and thus triggers the degradation of both MFNs, with a consequent mitochondrial fragmentation that favors mitochondria elimination^54–58^. Moreover, PINK1-phosphorylated MFN2 functions as a receptor for parkin, that, in turn, mediates MFN2 ubiquitination, as a signal to mark damaged mitochondria^59^. The ubiquitylation of mitochondrial surface proteins detaches mitochondria from microtubules and brings in mitophagy-initiating factors (reviewed in ref. 60). MFN2-KO in different cell types, such as MEFs, cardiomyocytes, dopaminergic neurons, suppresses mitophagy due to the impaired parkin translocation to mitochondria, resulting in damaged mitochondria accumulation and pathological conditions (see below). Similarly, age-related MFN2-depletion in muscles has been associated to an inhibited mitophagy and accumulation of dysfunctional mitochondria, linked to sarcopenia^61^.

### Axonal transport of mitochondria and other functions

MFN2 has been proposed to be essential for the transport of mitochondria along axons, being involved in their attachment to microtubules through interaction with the two main motor proteins Miro and Milton^62^. The phenomenon is not related to MFN2 activity on mitochondrial fusion, since an impaired transport is observed in MFN2-KO, but not in OPA1-defective neurons, though the ablation of these proteins induces similar mitochondrial fragmentation.

Several other intracellular pathways, such as cell cycle progression, maintenance of mitochondrial bioenergetics, apoptosis, and autophagy, have been demonstrated to be modulated by MFN2. Few of these aspects will be briefly discussed in the next sections. The readers are also referred to a recent review^63^.

## MFN2 and diseases

The importance of a regulated mitochondrial morphology in cell physiology makes immediately clear the potential impact of MFN2 in the onset/progression of different pathological conditions. Below, we summarize the main findings reporting a link between alterations in MFN2 and several diseases. A more detailed discussion is provided for CMT2A, i.e., a disease directly due to mutations in the *Mfn2* sequence.

### Charcot–Marie–Tooth disease type 2A (CMT2A)

Among different cell types, neurons are particularly sensitive to MFN2 defects: to work properly, these cells need functional mitochondria located at specific sites, i.e., dendrites and synaptic termini, to support adequate ATP production and Ca^2+^ buffering^64^. Indeed, *Mfn2* mutations are linked to neurological disorders characterized by a wide clinical phenotype that involves the central and peripheral nervous system^65,66^. The impairment of the former is rarer while neuropathy forms are more frequent and severe, involving both legs and arms, with weakness, sensory loss, and optical atrophy^65^. All these complex phenotypes are clinically collected in the neurological disorder CMT2A, a subtype of a heterogeneous group of congenital neuromuscular diseases which affect motor and sensory neurons, called CMT disease^67,68^.

**Fig. 5:**
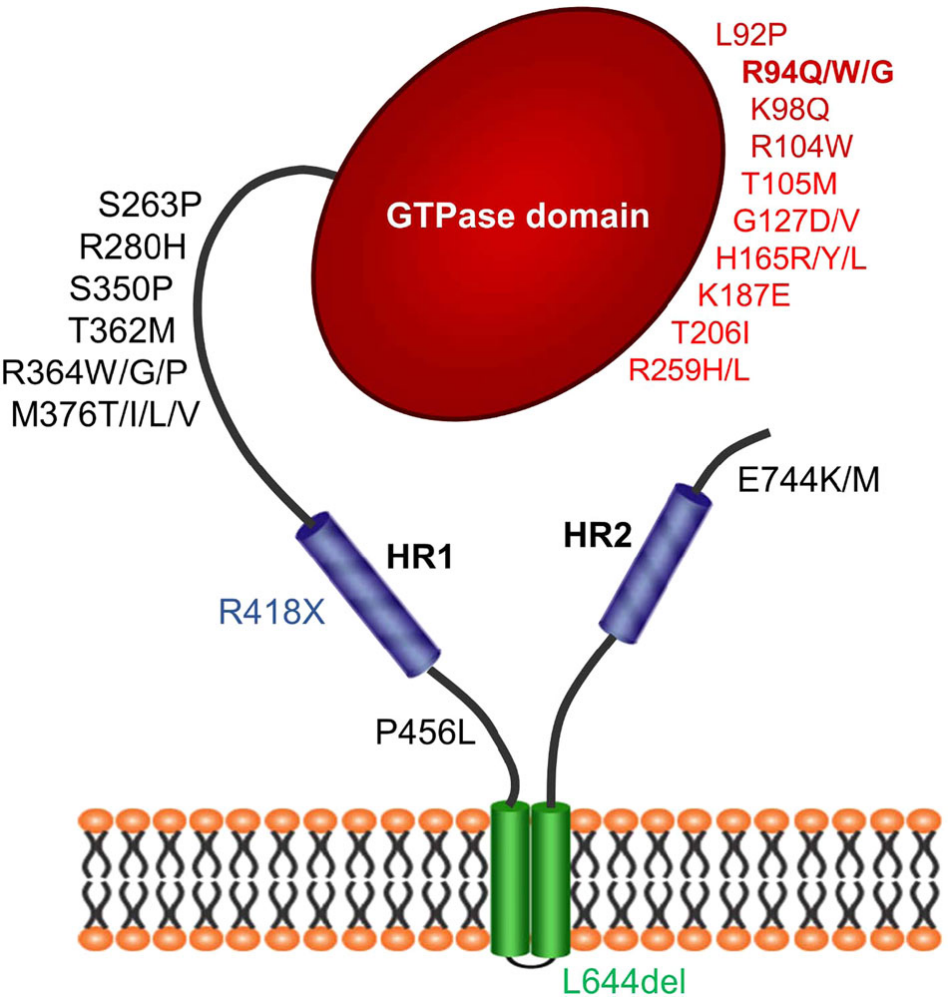
Schematic representation of MFN2 with its most common mutations linked to CMTA2 The scheme represents MFN2 protein and depicts amino acid mutations associated to CMT2A. The color refers to the domain affected: red, GTPase domain; blue, HR1/2; green, TM domains; black, linker regions. In bold, the more frequent mutations involving the arginine (R) in position 94

More than 100 dominant mutations in the *Mfn2* gene have been reported in CMT2A patients, the majority of which are missense mutations located in critical protein regions, particularly close to or within the GTPase domain and the coil-coiled motifs^67,69,70^ (Fig. 5). The most frequently mutated amino acid within the MFN2 protein is an arginine in position 94, and two mutations on this codon have been reported multiple times in independent patients (R94W and R94Q)^71^. This residue is highly conserved from humans to *Caenorhabditis elegans* and is located immediately upstream of the GTPase domain, within a hotspot region for mutations^72^ (Fig. 5). Some *Mfn2* mutations are considered “gain-of-function” and induce mitochondria aggregation, while other mutants result in a “loss-of-function” associated with mitochondrial fusion impairment^72,73^. Nerve biopsies of CMT2A patients show predominance of chronic axonal degeneration^72–76^, although this event is not present in late-onset patients^74^. Some reports described mitochondrial defects, with accumulation at distal sites of abnormally shaped mitochondria with cristae alterations^72,73,77–79^. It is however difficult to find a clear genotype−phenotype correlation because frequently the same mutation can be associated with different symptoms and age of onset^74^. *Mfn2* mutations have also been detected in a hereditary motor and sensory neuropathy type VI case with optic atrophy^80^.

How mutations in *Mfn2* lead to CMT2A is still debated and several mechanisms have been proposed as key events, in accordance with the different roles played by MFN2 within the cell. Firstly, a defective mitochondrial fusion has been suggested to participate in the pathogenesis of CMT2A. Indeed, mutations in both MFN2 and OPA1 result in neuropathologies characterized by specific neuronal degeneration^81^. Altered mitochondrial fusion could be pathologically linked to unbalanced mtDNA maintenance, and distribution: indeed cells lacking MFN2, or depleted of OPA1, show significant amount of mitochondria devoid of mtDNA^29^. Since mtDNA encodes several proteins of the respiratory chain, mtDNA depletion leads to oxidative phosphorylation impairment which could be easily linked to neurodegeneration, especially of those neurons, such as Purkinje cells, that show low MFN1levels, insufficient to compensate the MFN2 deficit. Interestingly, in conditional MFN2-KO mice, cerebellar Purkinje cells were selectively degenerated, showing defective mitochondria distribution in dendritic branches, less spines, and decreased cytochrome-c oxidase activity^29^.

Another important cell feature altered in the presence of MFN2 mutations is mitochondrial transport and indeed current models propose this defect as the major cause of CMT2A. An abnormal transport of mitochondria through the microtubule system can explain why mutations in a ubiquitously expressed protein, such as MFN2, lead, in CMT2A, to a selective vulnerability of particular cell populations, i.e., motor and sensory neurons. Moreover, it can lead to distal axonal degeneration and explain the typical peripheral CMT2A neuropathy, with longest axons firstly and mainly affected^82^. Multiple evidence sustains a key role of MFN2 in mitochondrial axonal transport: (i) rat dorsal root ganglia neurons expressing different disease-associated human MFN2 mutants show an altered mitochondrial axonal transport^83^; (ii) MFN2 interacts with the Miro/Milton complex and is required for organelles axonal transport^62,82^; (iii) both drug-induced MFN2 loss-of-function^84^ and MFN2 mutants-based^85^ CMT2A zebrafish models display a defective mitochondrial axonal transport; (iv) drosophila larvae deprived of Marf show defects in axonal mitochondria distribution^86^. Notably, both the disruption of proper mitochondria positioning along axons and the consequent axonal degeneration caused by CMT2A-related MFN2 mutations can be rescued by increasing the expression of MFN1, suggesting a certain grade of redundancy between the two proteins also in this function^82^.

Alterations in mitochondrial transport and distribution likely cause a bioenergetics impairment, especially in highly metabolic cells such as neurons. In a MFN2-R94W knock-in mouse model, however, heterozygous mice demonstrated decreased open-field activity, supporting a mild peripheral neuropathy, but did not exhibit deficits in axonal mitochondrial motility^87^. Similarly, spinal cord motor neurons derived by CMT2A patient inducible pluripotent stem cells showed changes in electrical properties but only mild axonal transport abnormalities^88^.

Thus, other neuronal functions are probably altered in the presence of MFN2 mutations. For example, in CMT2A fibroblasts carrying mutations in MFN2 (R364Q and A166T), defective mitochondrial bioenergetics have been reported, with reduced mitochondrial membrane potential and increased basal oxygen consumption and proton leak^89^. Accordingly, in vitro experiments using different cell models show that genetic modulation of MFN2 levels causes a deregulation of different metabolic pathways, changing mitochondrial membrane potential, oxygen consumption, and glucose oxidation^90–92^. Moreover, due to its involvement in mitophagy^55^ (see above), MFN2 mutations induce an impairment in mitochondrial turnover, leading to an accumulation of damaged organelles that impacts on neuronal homeostasis^93^. As a general note, being MFN2 an essential player in almost all mitochondrial dynamics, in the presence of mutations the alteration of all these aspects can differently contribute to the chronical axonal degeneration that characterizes CMT2A.

## Alzheimer’s disease

Increasing evidence suggests a possible link between MFN2 deregulation and Alzheimer’s disease (AD). In particular, MFN2 protein and mRNA levels are decreased in the frontal cortex of patients with AD^94^, as well as in hippocampal neurons of post-mortem AD patients^95^. Notably, the cortex and hippocampus are the brain’s areas in which a major neuronal impairment is observed in AD. On the same line, MFN2 is downregulated in primary hippocampal neurons from triple transgenic (3×Tg) AD mice^96^. Recently, in the senescence-accelerated mouse-prone 8 (SAMP8) line, a mouse model recapitulating the symptoms of late-onset sporadic AD, an age-dependent decrease in MFN2 expression in the hippocampus was found to be due to an increased expression of miR-195^97^. miR-195 binds the 3′-untranslated region of *Mfn2* mRNA, affecting MFN2 expression. Moreover, a significant correlation between the rs1042837 single nucleotide polymorphism in the *Mfn2* gene and AD risk has been found in Korean patients^98^. Interestingly, the *Mfn2* gene is located on chromosome 1p36, which has been suggested to be an AD-associated locus^99^.

If, however, MFN2 alterations are causative for the pathology or just a consequence of AD onset is currently unknown. In particular, it is not clear whether the defective MFN2 expression is linked to AD through its effects on mitochondrial morphology or by affecting additional pathways. Indeed, postmortem analysis of cerebella from PS1-E280A patients displayed impaired ER−mitochondria tethering, although MFN2 protein levels showed no significant alterations^100^. Recently, in different AD models, an increased ER−mitochondria coupling has been observed before the onset of clear disease hallmarks^101–104^. In particular, Presenilin-2 (PS2), one of the three proteins whose mutations have been associated to familial forms of AD (FAD), has been demonstrated to directly increase ER−mitochondria physical and functional tethering^101^, by binding and sequestering MFN2^41^. Interestingly, FAD-PS2 mutants, compared to the wt protein, are more potent in this function^101^, likely because of their enrichment at MAM, where they can more easily encounter and sequester MFN2^41^. Recently, an increased ER−mitochondria connection, induced by MFN2-downregulation in HEKs-APPswe cells, i.e., HEK293 cells overexpressing the FAD Swedish mutation in amyloid precursor protein, has been demonstrated to deeply affect the process of amyloid beta (Aβ) production^38^. Aβ peptide, and in particular the ratios between its slightly differently-long species, is thought to be critical in AD onset/progression, because it is the principal constituent of the amyloid plaques observed in the brains of AD patients. Specifically, the reported effect has been associated to an impaired maturation of γ-secretase^38^, a key enzymatic complex responsible for Aβ production. Moreover, Aβ-induced decreased MFNs levels have been observed in brains of AD-mice models^105^ and in a neuroblastoma cell line^106^. Importantly, in the latter study, MFN2, but not MFN1 overexpression, was shown to limit the Aβ-mediated neuronal cell death. In conclusion, the observed increased ER−mitochondria coupling in AD, and its possible link with decreased MFN2 levels, appears of particular interest for future investigations.

## Parkinson’s disease

As discussed above, MFN2 is a key substrate of the PINK1/parkin couple, whose mutations are linked to the familial forms of Parkinson’s disease (PD). MFN2, but not MFN1, has been demonstrated to be essential for axonal projections of midbrain dopaminergic (DA) neurons that are affected in PD^107^. Notably, parkin translocation to mitochondria in MFN2-KO DA neurons is impaired, with accumulation of abnormal mitochondria^107^. PD-linked mutations in PINK1 and parkin impair MFNs ubiquitination in human fibroblasts from patients, increasing mitochondrial branching. In drosophila, parkin overexpression reduces Marf levels, rising PGC-1α expression, promoting mitochondrial respiration and extending lifespan^108^. It is, however, difficult to exactly understand the role of the PINK1/parkin-MFNs axis in the progression of PD. For instance, MFN2 is specifically increased in MAM of fibroblasts from parkin-KO mice and from human PD patients with parkin mutations^109^. This MFN2 abundance has been associated to an higher ER−mitochondria coupling^109^. On the same line, activation of ER stress has been described in *pink1* and *parkin* mutant flies. The phenotype has been ascribed to the presence of Marf bridges, occuring between ER and defective mitochondria. Indeed, reducing Marf is neuroprotective, independently of the persistence of defective mitochondria^110^. On the other hand, co-expression of both PGC-1α and parkin in neurons has been shown to reduce MFN2 levels (by increasing protein’s turnover), to ameliorate mitochondrial respiration, to protect nigral DA neurons from mitochondrial damage and to increase ER−mitochondria coupling^111^. To further characterize the impact of MFNs alterations in the progression of PD, considering the capacity of PINK1 and parkin to trigger post-translational modifications in their substrates, we believe that not only the total levels of MFNs, but also the evaluation of the functional significance of these modifications could be of particular interest for future investigations.

## Obesity/diabetes/insulin resistance

In obesity and type II diabetes, MFN2 expression has been found to be reduced^90,112^. In turn, MFN2 downregulation (likely by increasing reactive oxygen species production and reducing mitochondrial respiration) activates JNK pathway, favoring the formation of lipid intermediates that lead to insulin resistance (IR) in both skeletal muscle and liver (reviewed in ref. 63).

There is general consensus in literature that MAM play a key role in cell metabolism. However, despite the role of MFN2 in obesity and IR, either increased^113^ or decreased^114,115^ ER−mitochondria association have been found in different obesity/IR mouse models (reviewed in ref. 36).

Recent studies in liver and adipose tissues of genetically or diet-induced obese mice have revealed increased UPR^116^ with defects in lipid biosynthesis and Ca^2+^ homeostasis proposed as causative. MFN2, by influencing ER−mitochondria crosstalk, has been suggested to play a role in this context. Indeed, liver-specific ablation of *Mfn2* in mice led to metabolic abnormalities, including glucose intolerance and enhanced gluconeogenesis. Interestingly, impaired insulin signaling and glucose tolerance are ameliorated by chemical chaperones or treatment with the antioxidant *N*-acetylcysteine^51^. Moreover, hypothalamic ER stress has emerged as a causative factor for the development of leptin resistance. Proopiomelanocortin neurons-specific ablation of *Mfn2* resulted in ER stress-induced leptin resistance, hyperphagia, reduced energy expenditure, and obesity. Again, pharmacological relieve of hypothalamic ER stress reversed these metabolic alterations^117^. Altogether, these data establish that MFN2-dependent ER stress has a crucial role in systemic and local energy balance. The regulation of this process appears very complex: indeed, a lipotoxic insult induced by saturated lipids decreases MFN2 expression, leading to ER stress response and IR in hypothalamic-derived cells. The same result is observed in arcuate nucleus of hypothalamus when lipotoxic insult is induced by mice high-fat feeding. IR is prevented when cells are pre-incubated with the ER stress release reagent 4 phenylbutirate^118^.

## Cardiomyopathies

In heart, the embryonic combined *Mfn1/Mfn2* deletion is lethal after e9.5, while in adults it induces a rapidly progressive and lethal dilated cardiomyopathy^119^. The phenotype is due to impaired mitochondrial fusion, suggesting the importance of this process in normal cardiomyocytes functions. Similar conclusions have been reached upon post-natal *Mfn*s KO in cardiomyocytes, with an accumulation of dysfunctional mitochondria that leads to cardiomyopathy^120^. Contrasting results have been obtained by deleting the only *Mfn2* in adult cardiomyocytes. A modest cardiac hypertrophy, associated to a tendency of MFN2-deprived mitochondria to be enlarged, was observed by Papanicolaou et al.^121^, who reported an increased resistance to Ca^2+^-mediated cell death stimuli due to a delay in mitochondrial permeability transition. On the other hand, dilated cardiomyopathy was observed by Dorn and colleagues upon conditional MFN2-KO, associated to an impaired mitophagy with accumulation of dysfunctional mitochondria (see above and ref. 59). MFN2 overexpression has been shown to increase cardiomyocytes susceptibility to oxidative stress-mediated apoptotic stimuli, through inhibition of Akt signaling and activation of the caspase-9 pathway^122^. In flies, Marf deficiency induces cardiomyopathy associated to sarcoplasmic reticulum stress and mitochondrial fragmentation; interestingly, however, cardiac-specific expression of Xbp1, a transcription factor that activates genes important for protein folding and ER-stress rescue, does not recover the fragmented mitochondrial morphology but fully normalized the contractile performance of Marf-deficient hearts^123^. In conclusion, while it is undisputed the importance of MFN2 in cardiomyocytes physiology, clarification of whether its pro-fusion activity or other functionalities of the protein are involved will require further investigations.

## Cancer

In several types of cancer, profound alterations of mitochondrial morphology, and in particular an increased network fragmentation, have been observed. In most cases, these alterations have been associated to changes in cancer cells metabolism, with a switch from mitochondria oxidative phosphorylation to glycolysis, according to the Warburg effect. Mitochondrial fragmentation is usually associated with an imbalance of the Drp1/MFNs ratio (reviewed in ref. 4). For instance, decreased MFN2 levels have been reported in liver^124^, colorectal^125^, and lung^126^ cancers. Interestingly, the rescue of mitochondrial morphology, by recovering MFN2 expression^126^ or downregulating Drp1^127^, reduces cell proliferation and increases spontaneous apoptosis. Recently, reduced MFN2 levels have been associated to a poor diagnosis in breast cancer patients^128^. Notably, the increased viability of cells expressing lower amounts of MFN2 was associated to an increased, pro-survival mTORC2/Akt signaling, whose pharmacological inhibition suppresses MFN2-deficient tumor growth^128^. Overall, increasing evidence suggests that a fragmented mitochondrial network, frequently associated to reduced MFN2 levels, provide tumor cells with an advantage for their growth, though the precise reasons are unknown. Mitochondrial fragmentation has been demonstrated to be protective against Ca^2+^-dependent apoptosis, by preventing propagation of Ca^2+^ waves within their matrix, thus limiting mitochondrial Ca^2+^ overload^129^. Alternatively, the increased OMM curvature, observed upon MFN1-depletion, has been suggested to impair Bax insertion into the OMM, preventing Bax-induced apoptosis^130^. A more detailed discussion on these topics can be found in a recent review^4^.

## Conclusions

MFN2 is a versatile protein able to modulate several fundamental pathways within the cell. Together with its homolog MFN1, it has a role in mitochondrial fusion but, independently from it, MFN2 has also several non-fusogenic functions, such as those of modulating ER−mitochondria tethering, ER functionality, and cell metabolism. Among different diseases in which its dysfunction has been described, CMT2A is directly caused by *Mfn2* mutations, while in other cases altered expression levels or post-translational modifications have been observed. Overall, we believe that the investigation of the detailed molecular mechanisms involved in MFN2 action, as well as of the cooperation with additional players, will allow a more precise evaluation of the role of MFN2 in the pathogenesis of several disorders, offering novel pharmacological opportunities.
